# Neuroanatomy and function of human sexual behavior: A neglected or unknown issue?

**DOI:** 10.1002/brb3.1389

**Published:** 2019-09-30

**Authors:** Rocco S. Calabrò, Alberto Cacciola, Daniele Bruschetta, Demetrio Milardi, Fabrizio Quattrini, Francesca Sciarrone, Gianluca la Rosa, Placido Bramanti, Giuseppe Anastasi

**Affiliations:** ^1^ IRCCS Centro Neurolesi “Bonino Pulejo” Messina Italy; ^2^ Department of Biomedical, Dental Sciences and Morphological and Functional Images University of Messina Messina Italy; ^3^ University of Chieti Chieti Italy

**Keywords:** dopamine, limbic system, neurosexology, serotonin

## Abstract

**Introduction:**

Sexual desire, arousal, and orgasm are mediated by complex, yet still not fully understood, interactions of the somatic and autonomic nervous systems operating at the central and peripheral levels. Disruption of endocrine, neural, or vascular response, caused by aging, medical illness, neurological diseases, surgery, or drugs, can lead to sexual dysfunctions, thus significantly affecting patients' quality of life.

**Purpose:**

This narrative review aims at characterizing the involvement of the central nervous system in human sexual behavior.

**Methods:**

A literature search was conducted using PubMed in its entirety up to June 2018, analyzing the studies dealing with the neurobiological and neurophysiological basis of human sexuality.

**Results:**

Sexual behavior is regulated by both subcortical structures, such as the hypothalamus, brainstem, and spinal cord, and several cortical brain areas acting as an orchestra to finely adjust this primitive, complex, and versatile behavior. At the central level, dopaminergic and serotonergic systems appear to play a significant role in various factors of sexual response, although adrenergic, cholinergic, and other neuropeptide transmitter systems may contribute as well.

**Conclusions:**

Providing healthcare professionals with information concerning sexual behavior may overcome useless and sometimes dangerous barriers and improve patient management, since sexual well‐being is considered one of the most important aspects of one's quality of life.

## Graphical abstract

Sexual desire, arousal, and orgasm are mediated by complex, yet still not fully understood, interactions of the somatic and autonomic nervous systems. At the central level, dopaminergic and serotonergic systems appear to play a significant role in various factors of sexual response. Providing healthcare professionals with information concerning sexual behavior may overcome useless and sometimes dangerous barriers.

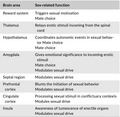

## 1 INTRODUCTION

Sexual response is a deeply rooted key physiological condition found throughout the species. The study of sexuality in animals is a complex topic that could be approached from different perspectives, given that it relies on the interplay between nervous, endocrine, and genetic factors. In humans, sexual behavior is influenced by cultural settings requiring dynamic behavioral adaptation. Therefore, a multisystem interaction is necessary to guarantee both features and the more complex functions typical of humans. Neural structures engaged in sexual behavior are located throughout the nervous system, both in its central and in its peripheral divisions. Detection of multimodal sexual stimuli involves sensory processing that merges with experiences to trigger autonomic as well as proper motor responses under an overwhelming cognitive control. To date, although the neural mechanisms underlying desire, arousal, and orgasm are the same in both males and females, sexual responses, however, are different between genders (Balthazart, 2016; Clark & Hatfield, 1989; Petersen & Hyde, 2010).

Such difference in sexual attitude between males and females can be partly attributed to dimorphic anatomical substrates located in the genital and nervous systems together with different hormonal profiles (Hausmann, 2017; MacLusky & Naftolin, 1981; McEwen & Milner, 2017). However, although several neuroimaging studies have shed new light on the mechanisms underlying sexual behavior in humans, some pieces of the puzzle are still missing.

According to Masters and Johnson, physiological responses during sexual stimulation consist of the sequential phases of excitement, plateau, orgasm, and resolution (Masters & Johnson, 1966). Later, Kaplan's triphasic model (consisting of three phases conceived as consequential and concatenated desire, excitement and orgasm) had a stronger appeal because of its clinical relevance (Kaplan, 1974).

Sexual desire is commonly defined as the presence of sexual thoughts, fantasies, and motivations to engage in sexual behavior in response to relevant internal and external cues (Buss & Schmitt, 1993). It is furthermore influenced by many factors such as attitudes, opportunity and/or partner availability, mood, and health. During the excitement phase, the body prepares for coitus, as the result of any erotic physical or mental stimulation leading to sexual arousal. Intimately connected with sexual desire, sexual arousal is defined in both subjective (i.e., feeling sexually excited) and physiological terms (i.e., genital vasocongestion and tumescence).

In males, physiological sexual arousal begins with an erection, which is a reflexogenic event driven by sensorial signals conveyed by dorsal nerve of the penis after stimulation of free nerve endings situated along the penis and glans. Penile hemodynamics during erection is characterized by tumescence of the cavernous bodies caused by vasodilatation. This is due to nitric oxide released by the endothelium after parasympathetic stimulation of pelvic nerves. On the other hand, penis detumescence is mediated by pelvic, cavernous, and pudenda nerves of the sympathetic nervous system together with several vasoconstrictor factors. Erection depends on spinal and supraspinal control in response to tactile, visual, imaginative, and olfactory inputs. It is likely that reflexogenic and psychogenic stimuli act synergistically via the sacral parasympathetic route. Although the supraspinal events involved in erectile function are poorly understood and mostly based on animal models, hypothalamic and limbic pathways seem to play a pivotal role in erection.

Arousal in women depends on similar mechanisms; however, sexual excitement is phasic with the menstrual cycle. Hemodynamics in the clitoris after sexual stimulation is controlled by the autonomic nervous system. During arousal, the Bartholin glands located on either sides of the vaginal opening produce mucus, which, together with vaginal secretion, lubricate the area in order to make sexual intercourse more comfortable (Yucel et al., 2004).

Furthermore, the excitement phase results in an increase in heart and breathing rates as well as in blood pressure, triggered by several nuclei, the brainstem, and hypothalamic medial preoptic area. Vasocongestion of the skin leads to sexual flush (mostly chest and neck), which usually disappears soon after orgasm occurs. Orgasm, the conclusion of the plateau phase, is characterized by quick jerky muscle contraction of the lower pelvic muscles surrounding the anus and primary sexual organs accompanied by an euphoric sensation and a further increase in heart rate (Masters & Johnson, 1966). Nonetheless, the apex of the arousal phase cannot be considered a simple physical sequence of happenings. In fact, it is widely known that sexual dysfunction (i.e., anorgasmia) seriously affects individuals' quality of life and psyche.

The aim of this scoping review is to provide healthcare professionals with useful information concerning human sexuality and its neural correlates in order to better manage their patients. Our main argument is that a better comprehension of human sexual behavior may offer a general perspective into (a) how the brain works to produce it in daily life for the fortunate, (b) how brain fails in the less fortunate, and hopefully (c) better habits to improve patients' quality of life.

## 2 METHODS

A literature search was conducted using PubMed (http://www.ncbi.nlm.nih.gov/pubmed) in its entirety up to June 2018. The search was done using different combinations of the following keywords: “sexual behavior,” “neurobiology,” “brain,” “neurotransmitters,” “dopamine,” “serotonin,” “central nervous system,” and “human sexuality.” Additional survey was carried out by screening references of the selected articles.

Moreover, we have used third part materials by our previous chapter on human sexuality (complying with the CC license under which the book was published), although the issue has been completely rebuilt and updated, also adding studies on female sexual function and behavior, and on brain functional neuroimaging (Calabrò & Bramanti, 2011).

## 3 NEUROANATOMY OF HUMAN SEXUAL BEHAVIOR

Sexual behavior relies on the processing of sexual stimuli, which allow individuals to enter the human sexual cycle. From an evolutionary point of view, this is a fundamental behavior, as it supports the interactions aimed at reproduction, which is critical for biological adaptation and species self‐preservation. However, sex impact in humans' everyday life lies broadly outside its archetypical purpose. Over the ages, several proofs have confirmed that a regular sexual activity positively influences both physical and psychic health. Therefore, it is important for physicians and other healthcare professionals to be updated on the neural mechanisms underlying sexual behavior. Each phase of human sexual cycle involves neural structures ranging from the cerebral cortex to the peripheral nerves. Sexual behavior in humans should be conceived as a pleasure‐seeking pulse that can be readily controlled in a context‐appropriate way under the influence of cultural factors such as moral and ethics.

Different unimodal specific sexual cues are processed in the central nervous system where complex integrative activities result in both autonomic and voluntary responses. The way sexual information flows through the brain reflects the scheme of goal‐directed behavior, namely the “sexual pleasure cycle” (Georgiadis & Kringelbach, 2012; Georgiadis, Kringelbach, & Pfaus, 2012). Sexual drive and pleasure experience are key components of the sexual pleasure cycle, whose experience depends on dopaminergic neurons of the reward system located mainly in the midbrain (substantia nigra pars compacta—SNc, ventral tegmental area—VTA) and the interacting opioid–endocannabinoid system.

Furthermore, sexual behavior requires implicit sensory stimuli that are evaluated as sexually salient and compared to past experiences, thus prompting an elicited motivational state. The respective anatomical substrates are limbic forebrain structures such as hypothalamus, amygdala, hippocampus, and nuclei of the septal region, employed in motivational states and emotional processing. Different sensory cues are integrated by limbic structures in an unconscious way, thus triggering typical autonomic responses (heart rate, blood pressure, and respiratory frequency increase along with brainstem structures). Nevertheless, all the stages of the human sexual cycle involve complex conscious awareness, which points at the cerebral cortex as the main character (see Table 1 for a summary of the brain structures involved in regulating human sexual behavior). A comprehensive review about the role of the cerebral cortex in sexual behavior is carefully described by Georgiadis (2015).

**Table 1:** Summary of the brain areas involved in human sexual behavior

Brain area	Sex‐related function
Reward system	Triggers sexual motivation Mate choice
Thalamus	Relays erotic stimuli incoming from the spinal cord
Hypothalamus	Coordinates autonomic events in sexual behavior Mate choice Mate choice
Amygdala	Gives emotional significance to incoming erotic stimuli Mate choice Modulates sexual drive
Septal region	Modulates sexual drive
Prefrontal cortex	Blunts the initiation of sexual behavior Modulates sexual drive
Cingulate cortex	Processing sexual stimuli in conflictuary contexts Modules sexual drive
Insula	Awareness of tumescence of erectile organs Modulates sexual drive

Therefore, the sensorimotor cortices are involved in triggering voluntary movements during sexual intercourse and in genital sensation, whereas higher order associative areas play a pivotal role in erotic mental imaginary and in the inhibition of sexual pulses. On the other hand, the spinal cord is mainly involved in penile and clitoral tumescence, vaginal and penile gland lubrication, and rhythmic contraction of perineum muscles. Other important areas such as the nucleus paragigantocellularis (nPG1), locus coeruleus (LC), raphe nuclei, and periaqueductal gray area, located in the brainstem, are intimately connected to the spinal cord and mainly involved in erection and ejaculation. Finally, with regard to the autonomic system, it should be noted that the parasympathetic system is implicated in erection and female lubrication, while the sympathetic one is involved in ejaculation and orgasm. Female lubrication occurs because of the interaction between neuropeptides involved in vessel muscular tone, namely the vasoactive intestinal peptide (VIP) that acts as vasodilator and the neuropeptide Y that induces venous vasoconstriction. This process leads to an increase in interstitial fluid, which results in vaginal lubrication (Levin, 2003).

### 3.1 The reward system

Analogous to other behaviors, sexual behavior has a beginning, a middle, and an end. All organisms that are engaged in sexual behavior share a common set of principles and endpoints that define the behavior itself, along with particular neural mechanisms that make it successful. To date, a behavior is defined as successful when it is flexible enough to maximize reward and minimize adverse outcomes.

Neural pathways that allow sexual response to become routine or automated with practice thus inducing plasticity and remodeling behavior are associated with positive sexual reinforcement, and they include dopaminergic‐releasing structures of the reward system. The aforementioned pathways have been demonstrated in animals and are likely to be present in humans as well, playing a decisive role in shaping individuals' behavior in an adaptive way in response to change in environment (Pfaus, Kippin, & Coria‐Avila, 2003).

In neuroscience, the reward system consists of the VTA, which is situated anteriorly with respect to the dorsal raphe nuclei and periaqueductal gray; besides, it has wide projections to the nucleus accumbens (NAc, ventral striatum) and to the prefrontal cortex. The first pathway constitutes the dopaminergic mesolimbic pathway, while the latter makes up the dopaminergic cortico‐limbic one. In addition, at the level of the midbrain, SNC dopaminergic neurons project mainly into the dorsal striatum. Neurons belonging to the reward system exhibit both a spontaneous tonic and a phasic activity. Spontaneous tonic spikes are thought to subserve basal level of extracellular dopamine, constituting a “background activity,” while phasic activity induces a sudden increase in dopamine levels, which have been associated with reward error predictions (Schultz, 2010). Since phasic burst mode firing relies on presynaptic activity, the acknowledgment of several structures projecting to VTA modulating its firing pattern through different neurotransmitters is noteworthy (Floresco, West, Ash, Moore, & Grace, 2003; Lecca, Melis, Luchicchi, Muntoni, & Pistis, 2012; Pignatelli & Bonci, 2015).

In particular, GABAergic neurons of NAc, ventral pallidum, and rostromedial tegmental nucleus modulate VTA neuron firing pattern, exhibiting a regulatory effect, which could shift tonic activity to burst mode. On the other hand, glutamatergic afferents (playing an excitatory role) from the prefrontal cortex, the bed nucleus, and pedunculopontine tegmentum do induce burst firing. Indeed, a recent fMRI study demonstrated that the OFC codes for both the value and the identity of reward, while activity of the vmPFC seems to be more involved in categorizing stimuli across reward categories (Howard, Gottfried, Tobler, & Kahnt, 2015). Therefore, rewards shape behavior since several cortical areas ascribe a value to incoming predictive stimuli leading to adaptive choices.

Reward processing is further modulated by the endocannabinoid system that produces lipoid neuromodulators whose specific receptors (CB1) have been found within the VTA, hippocampus, amygdala, and hypothalamus (for an extensive review of the influences of endocannabinoid system on behavior, see Sagheddu, Muntoni, Pistis, & Melis, 2015).

Several studies pointed out dopamine (DA) as the main actor in triggering sexual motivation, suggesting that the increase of DA levels in structures belonging to the reward system would lead the behavioral shift toward hypersexuality. It is worth noting that sexual stimuli are engaged by many neural substrates (i.e., NAc, caudate, insula, thalamus, orbitofrontal cortex—OFC, and dorsal anterior cingulate cortex—dACC) involved in reward processing, as well as in complex cognitive functions, such as decision‐making and salience (Haber, 2011; Haber & Knutson, 2009).

Aside from the well‐known role of the NAc (that is a neural structure of the ventral striatum) in the reward processing, various neuroimaging studies demonstrated an increased activity of the ventral striatum in response to erotic stimuli (Brand, Snagowski, Laier, & Maderwald, 2016; Childress et al., 2008). Notably, the NAc is part of a wide network of neural correlates of sexual arousal and it has been reported to track sexual preferences in heterosexual, homosexual, and bisexual individuals, both in men and in women (Safron et al., 2007, 2017, 2018). In addition, Oei, Rombouts, Soeter, Gerven, and Both (2012) showed that DA stimulates activity in the NAc and dACC, in response to subconsciously perceived sexual stimuli, thus suggesting the possibility for DA to influence sexual motivation at its earliest onset, which seems to occur outside awareness (Oei et al., 2012).

Considered as a major structure of the reward system, the thalamus has been traditionally conceived as a relay center of sexual stimuli from the spinal cord to superior cortical and subcortical structures. Electric stimulation of the thalamus and deep brain stimulation have been shown to influence penile erection respectively in primates (Robinson & Mishkin, 1968) and humans (Temel et al., 2004). Furthermore, several fMRI studies correlated increased thalamic activity to erotic visual stimuli (Park, Kang, et al., 2001; Park, Seo, et al., 2001; Redouté et al., 2000). Thalamus has been reported to subserve sexual preference processing and to relay them to the temporal lobes where they develop in complex behavioral changes. Thus, the thalamus is likely to play a pivotal role in mate choice (Mutarelli, Omuro, & Adoni, 2006; Spinella, 2004), reinforcing the idea that conceives the thalamus not only as a relay center from the spinal cord to the cortex and vice versa, but also as an integration hub that is active throughout the desire, arousal, and orgasm phases.

### 3.2 Subcortical limbic structures

#### 3.2.1 Hypothalamus

The hypothalamus is the ventral portion of the diencephalon lying below the thalamus, consisting of several nuclei with a variety of functions. The hypothalamus represents only 2% of brain volume, but it plays a crucial role in the integration of endocrine, autonomic, and behavioral responses (Braak & Braak, 1992).

Hypothalamic releasing factors in turn stimulate or inhibit the secretion of pituitary hormones, influencing body temperature, hunger, thirst, circadian cycles, and sexual drive (Sam & Frohman, 2008). showed that small lesions in the medial preoptic area/anterior hypothalamus (MPOA/AH) temporarily affected the sexual drive in rats, while larger lesions had permanent effects on sexual behavior. Although MPOA stimulation modulates erection and coordinates autonomic events associated with sexual response, the exact role of the MPOA/AH in sexual behavior has been controversial for a long time. Animal studies often reported contradictory results concerning the leading role of MPOA in sexual motivation and/or in sexual consummation (Davidson, 1966; Hughes, Everitt, & Herbert, 1990; Paredes, Tzschentke, & Nakach, 1998). It has recently been proposed that the MPOA is likely to receive information relayed from the hippocampus via the lateral septum and from the amygdala via the bed nucleus of the stria terminalis (BNST; Pfaus, 2009). Signals are further processed by the periaqueductal gray, which projects into the brainstem nuclei. The arrival of sexual stimuli to the MPOA triggers sexual motivation once they are further integrated with information relayed by the ventromedial, suprachiasmatic, infundibular, and ventral premammillary nuclei.

In a recent fMRI study, Brunetti et al. (2008) found a significant correlation between activation of bilateral hypothalamus and deep sexual identity (DSI), which is defined as a multidetermined constancy system combining biological, psychological, and cultural aspects of sexual identity (Brunetti et al., 2008). More specifically, DSI was assessed following the revalidation of the Franck drawing completion test introduced by Olivetti Belardinelli (Olivetti Belardinelli, 1994), resulting in a degree of concordance between self‐reported and psychological sexual identity, when subjects were exposed to erotic stimuli (Brunetti et al., 2008). Taking into account that DSI is accounted for interindividual differences in psychological view attitude to sex, the significant positive correlation between BOLD activity of bilateral hypothalamus and DSI (the higher the hypothalamus activation, the higher the concordance between self‐reported and male psychological identity—the personal self‐representation of being a male) indicates that the hypothalamus could be an anatomical substrate for individual differences in some features of sexual behavior. However, we consider this assumption as a mere hypothesis since the current literature lacks stronger evidences.

Moreover, the hypothalamus is involved in penile erection through two of its several nuclei, that is, the dorsomedial hypothalamic nucleus (DMHN) and ventromedial hypothalamic nucleus (VMHN). The DMN projects into the mesencephalic reticular formation via the central and dorsal gray matter and locus coeruleus. Both the DMN and VMN reach the lumbosacral autonomic centers involved in penile erection via the dorsolateral funiculus of the spinal cord. Interestingly, hypothalamic nuclei in turn receive information directly from the genital regions (Marson & McKenna, 1994; Rajaofetra et al., 1992; Sachs, 1995; Steers, 2000). In addition, it has been shown that the PVN (paraventricular nucleus) is activated during copulation and orgasm (Komisaruk & Whipple, 2005); this could be related to its specific endocrine function since it produces oxytocin, vasopressin, enkephalins, and dopamine (Argiolas & Melis, 1995). In particular, oxytocin has been demonstrated to trigger activity in structures belonging to the reward system after sexual‐ or social‐relevant stimuli (Gregory, Cheng, Rupp, Sengelaub, & Heiman, 2015; Groppe et al., 2013; Scheele, Plota, Stoffel‐Wagner, Maier, & Hurlemann, 2016; Scheele et al., 2013). Moreover, it has been demonstrated in rats that sensorial stimuli elicit activity especially on oxytocin‐secreting neurons (Yanagimoto, Honda, Goto, & Negoro, 1996). However, activation of PVN during fMRI does not mean co‐occurrence of oxytocin secretion, since fMRI is unable to identify oxytocin‐secreting neurons; thus, this hypothesis still remains speculative. Finally, the anterior hypothalamus has a role in the regulation of typical male sexual behavior. Indeed, it has been demonstrated that the interstitial nucleus of the anterior hypothalamus 3 (INAH3) is dimorphic with sexual orientation, in men and women, suggesting that sexual orientation has a biological substrate as it has been reported to be smaller in women and gay men (Brunetti et al., 2008; LeVay, 1991; Paredes & Baum, 1997; Swanson & Petrovich, 1998).

#### 3.2.2 Amygdala

The amygdala is an almond‐shaped group of nuclei located deep within the medial temporal lobes of the brain in complex vertebrates, including humans. Widespread connections of the amygdaloid complex with both cortical and subcortical structures underline that the amygdala plays an important role in human sexual drive (Baird, Wilson, Bladin, Saling, & Reutens, 2004; McKenna, 1999; Swanson & Petrovich, 1998). Indeed, bilateral lesions of the amygdala have accounted to be responsible for abnormal sexual behaviors (“hypersexed states”), such as those observed in Kluver–Bucy syndrome (Lanska, 2018).

For instance, the most ancient region of the amygdala, the cortical nucleus, is involved in the sense of smell and pheromone processing as it receives input from the olfactory bulb and olfactory cortex, thus playing a fundamental role in macrosmatic animals' sexual behavior. Amygdala is part of a large‐scale network of brain structures involved in emotion processing, a complex phenomenon that relies on decoding, integration sensorial stimuli, comparing this incoming flow of information to past experiences.

The amygdala acts as one of the main characters in the social brain (Adolphs, Tranel, & Damasio, 1998; Adolphs, Tranel, Damasio, & Damasio, 1994), a system accounted for behavioral shaping in context‐adaptive trend. After the stimuli gain an emotional relevance, they are conveyed from the amygdala to the prefrontal cortex and OFC. Moreover, the amygdala projects both to the previously mentioned structures fundamental for sexual behavior such as the hypothalamus and NAc. Thus, the amygdaloid complex provides regulation of autonomic responses and complex cognitive functions.

Findings from animal studies have clearly pointed out the amygdala as a key structure in mediating sexual behavior. In humans, evidences for the role of the amygdala in sexual functioning come from lesion studies, showing that stimulation of the amygdala evokes orgasmic‐like pleasure sensations (Baird, Wilson, Bladin, Saling, & Reutens, 2007). In addition, Baird and colleagues demonstrated that a significantly larger amygdala volume contralateral to the site of temporal lobe resection was associated with postoperative sexual improvement (Baird et al., 2004). One hypothesis that may partially explain this relationship emphasizes the involvement of the amygdala in the emotional stimuli processing. Indeed, the amygdala receives projections from the unimodal sensory areas and proceeds to a complex integration, which is associated with the assessment of emotional aspects.

Finally, many fMRI studies showed clear gender‐related differences in amygdala functioning. In fact, amygdala activation has been found to be increased in men compared to women even when the latter present greater sexual arousal (Hamann, Herman, Nolan, & Wallen, 2004; Seok, Sohn, & Cheong, 2016). Moreover, morphological and functional amygdalar differences have been demonstrated in homosexual individuals, thus supporting the hypothesis that sexual preference in humans is partly due to dimorphic structures (Poeppl, Langguth, Rupprecht, Laird, & Eickhoff, 2016). Greater preference‐related amygdala activity was observed in homosexuals compared with heterosexual men, but it is unclear whether this was a cause or a consequence of their sexuality (Safron et al., 2007). However, the same findings have not subsequently replicated (Safron et al., 2017). In addition, Wehrum et al. (2013) identified a common neural network playing a major role in processing sexual stimuli in men and women involving amygdala, insula, and thalamic activation regardless of the gender. On the other hand, apart from such similarities, overall stronger responses were reported in men, possibly reflecting stronger sexual responsivity of men than women (Wehrum et al., 2013).

#### 3.2.3 Cortical areas

**Figure 1:**
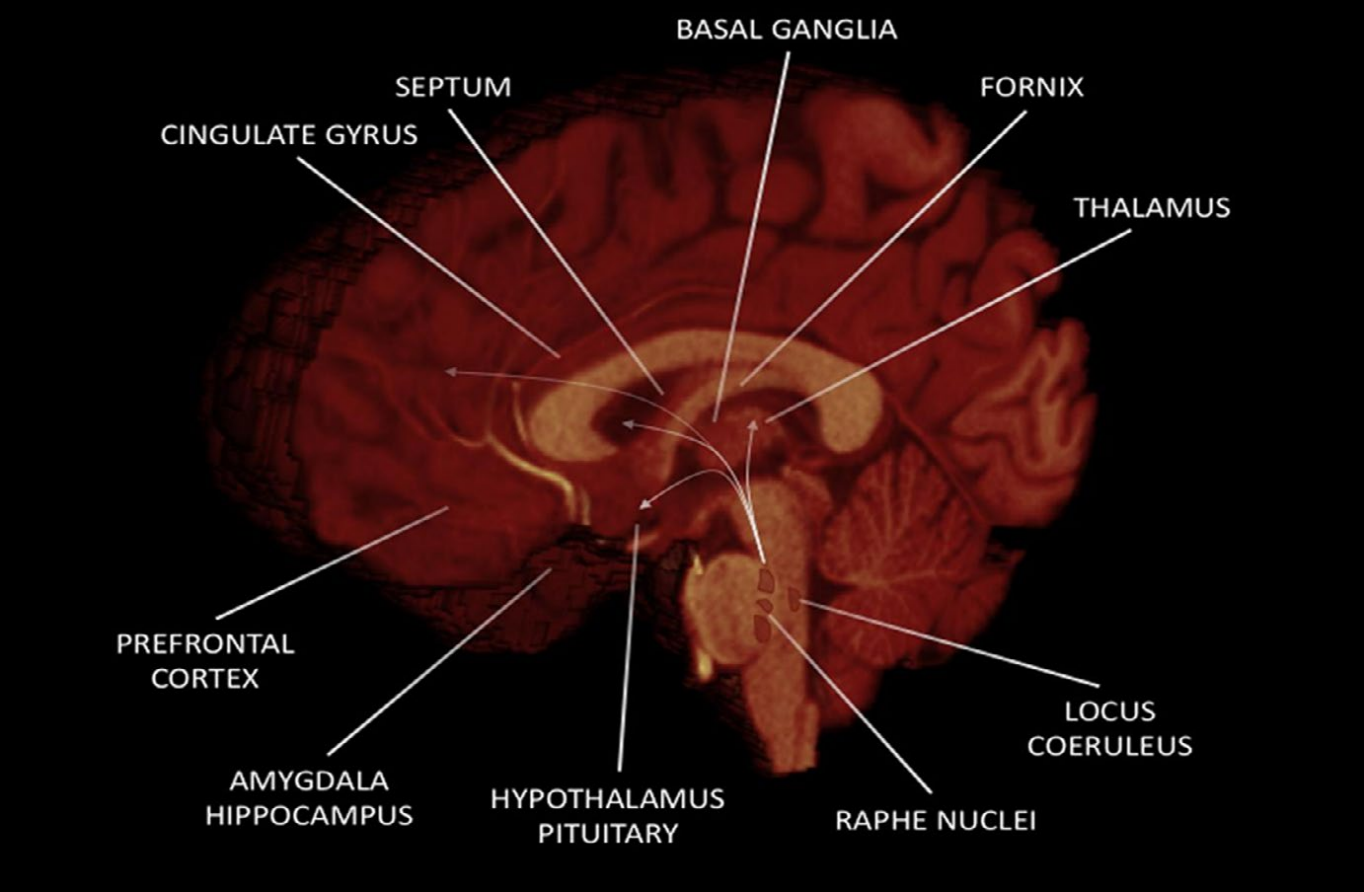
Main brain regions involved in human sexual behavior

Throughout evolution, sexual behavior has become increasingly complex, and although a substantial role is certainly to be attributed to subcortical structures of the limbic system and several nuclei of the brainstem, the cerebral cortex acts as a main character in making sexual behavior adaptive and shaped on social and cultural influences. Indeed, several cortical areas (Figure 1) are employed in the conscious processing of sexual stimuli and in computing the appropriate responses to sexual desire. Herein, we consider the role of the prefrontal cortex, OFC, cingulate cortex, and insula in human sexual behavior.

#### 3.2.4 Prefrontal and orbitofrontal cortices

The prefrontal cortex (PFC) is involved in planning complex cognitive behaviors, personality expression, decision‐making, and moderating correct social behavior (Amodio & Frith, 2006; Domenech & Koechlin, 2015; Frith & Dolan, 1996; Miller & Cohen, 2001). This brain region plays a pivotal role in shaping thoughts and actions in accordance with internal goals, in order to fully integrate and elaborate stimuli in the executive function outputs. Executive functions relate to the abilities to differentiate between conflicting thoughts, and to determine good and bad, better and best, same and different future consequences of current activities, working toward a definite goal, prediction of outcomes, expectation based on actions, and social “control” (the ability to suppress urges that, if not suppressed, could lead to socially unacceptable outcomes) (Kelly et al., 2004; Ridderinkhof, Wildenberg, Segalowitz, & Carter, 2004; Siddiqui, Chatterjee, Kumar, Siddiqui, & Goyal, 2008).

It is believed that sexual inhibition is an adaptive response that serves both reproductive and social endpoints (i.e., to keep individuals out of trouble or to allow a sufficient amount of sexual satiety appearing as a “refractory phase”). This should result in the inhibition of a complex and ongoing interplay of motor tendencies to planned and sustained actions. With regard to sexual behavior, it is believed that cultures superimpose a moral value of “right” and “wrong” on the hierarchies so that some “feeling‐good” behaviors are right and can be experienced without guilt, whereas others are wrong and carry the weight of guilt and/or of rules of law against them. Thus, this type of inhibition represents an approach–avoidance conflict, where the expectation of reward drives the desire, but the real or perceived aversive consequences of engaging in sexual activity blunt the initiation of behavior (Berkman & Lieberman, 2010; McNaughton, 2010). Moreover, sexual inhibition can also be induced by sexual nonreward suppressing desire components. Accordingly, the “prosexual” nature of drugs such as alcohol or cocaine may act through the ability to disinhibit such suppressed sexual responding. These inhibitory systems are located in the PFC and may inhibit the activation of excitatory mechanisms shifting attention and behavior to nonsexual stimuli or situations. Opioids, which mediate sexual reward states, endocannabinoids, which induce sedation, and serotonin, which induce satiety, seem to be, at least, the three neurochemical systems involved in sexual inhibition (Pfaus, 2009).

Hypersexuality, conceived often as disinhibited sexual behavior, has been observed in patients with OFC lesions. However, data from clinical studies and case reports are not totally convergent. In addition, lesions occurring strictly within the OFC are not so recurrent. Thus, disinhibited sexual behavior is referred to lesions in the frontal lobe, which would impair the inhibitory processes arising from these regions (Baird et al., 2007; Béreau, 2018). Furthermore, OFC regions have been addressed for pleasant body representation and euphoric feelings reached at the peak of sexual cycle. Finally, both PFC and OFC are deeply connected to subcortical structures belonging to reward system and are responsible for the cognitive filtering of sexual behavior (Schmidt et al., 2017).

### 3.3 Cingulate cortex

Cingulate cortex is the most conspicuous part of the limbic system, and it surrounds the corpus callosum from the rostrum to its splenium. It is divided into anterior, a middle, and a genual subregion. The anterior cingulate cortex (ACC) is further divided into two functionally segregated areas: ventral and dorsal (vACC/dACC). Many studies point out the role of the ACC in sexual behavior. fMRI studies reveal ACC activation in response to erotic stimuli, suggesting that ACC is involved in processing sexual stimuli in divergent contexts, enhancing decision‐making through its outputs to motor‐related areas and periaqueductal gray (Arnow et al., 2002; Rauch et al., 1999; Redouté et al., 2000; Seok et al., 2016). In addition, Oei et al. (2012) showed increased dACC activation due to DA release when sexual stimuli were presented out of awareness, thus indicating its involvement in the subliminal stimuli processing (Oei et al., 2012).

On the other hand, activity in the middle cingulated cortex has been reported to be elicited during arousal, while activity in the subgenual cingulate cortex seems to negatively correlate with arousal (Stoléru, Fonteille, Cornélis, Joyal, & Moulier, 2012). The white matter adjacent to the subgenual cingulate region represents a critical target for deep brain stimulation in treatment of resistant depression, whose activity is pathologically elevated, inducing signs of remission in most of patients (Mayberg et al., 2005). Finally, increased cingulate cortex activation was observed in males during erection and in females during orgasm (Schober & Pfaff, 2007). Therefore, taken together these findings suggest that this cortical region is involved in several features of sexual behavior, being a key relay structure between subcortical limbic structures and associative cortices.

#### 3.3.1 Insula

As previously introduced, sexual behavior complexity is mainly related to its conscious modulation. The insular cortex is a hub of the salience network (Uddin, 2014; Wager & Barrett, 2004), processing sensorial stimuli and relying them to other cortical areas, thus facilitating attention and working memory. Several sex‐related task fMRI studies demonstrated the co‐activation of the insula and ACC, suggesting that these cortical areas are somehow coupled in their functions (Oei et al., 2012). Similar to the cingulate cortex, insula conveys integrated information to brainstem structures in order to regulate autonomic responses and, at the same time, to the motor‐related area triggering appropriate responses to sexual stimuli (Both et al., 2008; Janssen, Everaerd, Spiering, & Janssen, 2000).

It has been found that the anterior insula is mainly active during the desire phase, while during the arousal phase, the activity is shifted in the posterior part of the insula (Georgiadis, 2015). Taking into account that mesolimbic dopaminergic projections reach both the insula and ACC as well as the NAc, the functional coupling between these cortical areas in processing rewarding sex‐related stimuli is further reinforced. Disorders in genital arousal have been reported in a fMRI study conducted on patients affected by symptomatic epilepsy due to insula lesions (Anzellotti et al., 2010).

According to Eickhoff et al. (2010), body maps would be computed in the posterior part of the insula, suggesting a possible role in processing haptic erotic stimuli. The position of the genitals in these maps is still to be determined (Eickhoff et al., 2010). The representation of genitals in the somatosensory cortices in humans has been recently subject of a debate. Activation of the genital region of the primary somatosensory cortex and secondary somatosensory cortex has been demonstrated after tactile self‐stimulation of genitals (Wise, Frangos, & Komisaruk, 2016). The position of both male and female genitals in the so‐called “genital cortex” has been also recently described in rats (Lenschow et al., 2016). For a more extensive view on the somatosensorial representation, the systematic review by Cazala and colleagues is recommended (Cazala, Vienney, & Stoléru, 2015). Moreover, the insula region has been employed in making individuals aware of tumescence and engorgement of erectile organs during sexual arousal, with a far greater activation of insula in men (Rupp & Wallen, 2008).

## 4 THE HUMAN SEXUAL BEHAVIOR FROM A NETWORK PERSPECTIVE

The last decades have been characterized by a paradigmatic shift in the field of human brain mapping, allowing to investigate human brain structure and function not only at the level of single areas, but also from a network perspective.

Indeed, according to the recent *associationist* theory, the brain consists of several, segregated and parallelly distributed networks around critical and participating cortical epicenters (Catani & Thiebaut de Schotten, 2012; Mesulam, 2000; Zappalà, Thiebaut de Schotten, & Eslinger, 2012) that allow a good trade‐off between cost and efficiency in information transferring.

Neuroimaging studies have demonstrated that human sexual response involves a variety of cortical and subcortical brain areas, showing very similar activation patterns across gender and sexual preferences.

Georgiadis and Kringelbach (2012) identified (a) a “sexual wanting pattern” mainly including the superior parietal lobule, the temporo‐occipital areas, NAc, OFC, ACC, amygdala, and hippocampus and (b) a “sexual liking pattern” involving the inferior parietal lobule, hypothalamus, insula, ventral premotor cortex, and the middle cingulate cortex (Georgiadis & Kringelbach, 2012). Abnormal activation patterns in the sexual wanting network have been linked to patients with inappropriate sexual behaviors and hypersexuality (Kühn & Gallinat, 2016; Voon et al., 2014); on the other hand, negative correlation patterns have been demonstrated between sexual cue‐induced brain activity and hypersexuality severity (Klucken, Wehrum‐Osinsky, Schweckendiek, Kruse, & Stark, 2016). By contrast, sexual interest disorders have been found to be associated with reduced sexual cue sensitivity, as suggested by structural and functional changes in sexual wanting‐related areas such as the amygdala, ACC, and NAc (Banca et al., 2016). Liking sex, instead, involves the recruitment of a brain network that is relatively distinct from the sexual wanting network. Connectivity and gray matter alterations within the brain areas belonging to the sexual liking network have been observed in patients with psychogenic erectile dysfunction. Indeed, different fMRI studies show altered connectivity between the insula and the ventral premotor areas, suggesting a possible aberrant inhibition control. More recently, Zhao et al. characterized the whole‐brain network topology of psychogenic erectile dysfunction patients compared to healthy controls, showing a similar small‐world organization (Zhao et al., 2015). However, at a closer look, psychogenic patients' network topology showed an unbalanced trade‐off between local specialization and global integration toward the former, thus resulting in a reduced overall global integration of information transferring.

Behaviorally speaking, the brain continuously acts to maintain a balance between networks that promote approach and networks that promote avoidance. It seems that the prefrontal areas show hyperactivation in patients with hyposexual behavior, although opposite results have been found in breast cancer survivors. Indeed, Versace et al. (2013) administering pornographic pictures to these patients demonstrated a reduced activity in the PFC and ACC, suggesting that chronic stressors might be related to a top‐down regulation of the PFC on cortical and subcortical structures in the human sexual behavior network (Versace et al., 2013).

The novel and rapidly evolving framework of network neuroscience has been reinforcing the idea that sexuality is a complex concept relying on the strict structural and functional interplay between (also) spatially remote brain areas that cooperate with each other in order to guarantee human sexual pleasure cycle.

## 5 SEXUAL DESIRE AND SEXUAL AROUSAL

Sexual desire or libido is defined as the broad interest in sexual objects or experiences, while sexual arousal is both a subjective (i.e., feeling sexually excited) and a physiological (i.e., genital vasocongestion) term. While sexual hormones have a critical role in modulating sexual arousal, sexual desire in humans seems to be initiated by the reception/ perception of sexual pheromones (Motofei, 2009), which are substances secreted by glands in the anus, urinary outlet, breasts, and mouth (Motofei, 2009; Motofei & Rowland, 2005).

The mechanisms underlying generalized arousal are complex and involve many cerebral circuits (Devidze, Lee, Zhou, & Pfaff, 2006). The ascending pathways have five major neurochemical systems that contribute to the arousal of the forebrain, that is, those signaled by norepinephrine, DA, serotonin, acetylcholine, and histamine, while the role of glutamate is less widely recognized.

Reticular neurons along the ventral and medial borders of the medullary and pontine reticular formation are important for the regulation of central nervous system arousal, which is as they respond to pain, genital sensation, to CO_2_ levels in the blood, and to changes in body temperature and cardiovascular functions. Other important axons descend from the paraventricular nucleus and from the preoptic area of the hypothalamus affecting the arousal aspects.

A neurobehavioral and multifaceted model of neural mechanisms for sexual arousal that includes a cognitive, an emotional, a motivational, and an autonomic component has been proposed by Ernst et al. (Ernst, Pine, & Hardin, 2006).

The cerebral areas linked to the cognitive mechanism of sexual arousal include the “attentive” network relaying in the OFC and the superior parietal lobules, while the motivational component is believed to be stored in the caudal part of the ACC, related to motor preparation processes; finally, the autonomic mechanism would involve the hypothalamus, insula, and the rostral part of the ACC (Ferretti et al., 2005).

In particular, the neural pathways of sexual arousal due to visual stimuli have been identified (Ferretti et al., 2005). This circuit includes limbic (hypothalamus, hippocampus, and amygdala) and paralimbic areas (ACC, frontal lobe, and insula), the associative cortices (inferior temporal and occipital cortices), and other subcortical and cortical sensory relays (thalamus and secondary somatosensory cortex—SII) (Calabrò & Bramanti, 2011; Ferretti et al., 2005).

It can be hypothesized that the autonomic and endocrine control of sexual behavior is mediated by the hypothalamus, while the activation of the amygdala is related to the appraisal process through which erotic stimuli are evaluated as sexual enticement. Indeed, the amygdala partakes in the evaluation of the emotional content of the complex perceptual information associated with the visual processing of the erotic stimuli (Sennwald et al., 2016).

The insula seems to be linked to the activation of the somatosensory processing pathway. The activation in this area, together with the thalamic and SII activation, may therefore reflect the participant's perception of his own behavioral response (Calabrò & Bramanti, 2011). Finally, the ACC and the PFC play a role in the evaluation of the motivational/emotional information and in the initiation of goal‐directed behavior, since these areas are specifically related to the monitoring and the control of emotionally driven behaviors (Bush, Luu, & Posner, 2000).

Although most of the data come from animal models, olfactory stimuli play an important role in sexual arousal: The specific anatomical pathway involves the rhinencephalon with regard to the cingulated gyrus, septum, and hippocampus (Snowdon, Ziegler, Schultz‐Darken, & Ferris, 2006).

## 6 NEUROBIOLOGY OF SEXUAL FUNCTION

Within the past decade, research has directed increasing attention to the neurobiology of sexual function. This has been fostered by growing awareness of the deleterious effects of pharmacological agents on sexual behavior, by an increased recognition of the high incidence of male sexual problems, and by the enormous success of phosphodiesterase inhibitor use for the treatment of erectile dysfunction. In this section, we provide a brief report of the role played by the most important endocrine and neurotransmitter factors in sexual function (see Table 2 for a summary) (Calabrò & Bramanti, 2011).

**Table 2:** Summary of the main neurotransmitters and modulators involved in regulating human sexual behavior

Neurotransmitters and modulators	Function in regulating human sexual behavior
Serotonin	Mainly released by the neurons of the raphe nuclei, serotonin acts on the smooth muscles of the vascular system of the genitals and other sexual organs to produce vasoconstriction and vasodilatation. At the central level, it instead has an inhibitory role on erectile function, lubrication, and sexual interest.
Dopamine	Striatal dopamine is important for motor aspect of copulation, but not for sexual motivation. Depending on its concentration, dopamine in MPOA disinhibits genital reflexes (low levels), facilitates parasympathetically mediated erections and copulatory behavior (moderate levels), and promotes sympathetically mediated ejaculation but inhibits erections (high levels).
Norepinephrine	It stimulates penile erection via autonomic activation and can reverse the sexual inhibition that follows sexual exhaustion, thus being useful in the treatment of erectile dysfunction and anorgasmia.
Acetylcholine	It is implicated in penile erection, and it has been shown to be useful in reversing antidepressant‐induced erectile and ejaculation difficulties.
Histamine	At the peripheral level, histamine leads to the full or partial erection via the activation of H2 and H3 receptors. At the central level, it instead modulates sexual behavior and libido.
Opioids	The tight interplay between opioids and hormones such as LH and testosterone consequently leads to sexual impairment. In particular, increased activity of the opioids, paralleled by a reduction of the levels of LH and testosterone, causes loss of libido, erectile dysfunction, and inability to reach orgasm.
Sex hormones	Androgens play a key role in both stimulating and maintaining sexual function in man, being critical for penile tissue development, growth, and maintenance of erectile function. Estradiol is responsible for the behavioral development of male mammals, acting either by increasing or decreasing male‐typical behaviors. In addition, sexual hormones seem to play an important role in sexual arousal by ensuring cerebral integration between somatic and autonomic sexual systems. Finally, prolactin provides the body with sexual gratification after sexual acts, although high blood levels of prolactin are likely to produce impotence and loss of libido.

### 6.1 Serotonin

Serotonin is a monoamine neurotransmitter, found extensively in the gastrointestinal tract of animals. About 80%–90% of the total serotonin in the human body is located in the enterochromaffin cells of the gut where it is used to regulate intestinal movements (Berger, Gray, & Roth, 2009). The remaining serotonin is synthesized in serotonergic neurons in the central nervous system, where it has various functions, including the regulation of mood, appetite, sleep, muscle contraction, and some cognitive functions including memory and learning. Human neurons of the raphe nuclei are the principal source of 5‐hydroxytryptamine (5‐HT) release in the brain (Calabrò & Bramanti, 2011). Axons of neurons in the *caudal* raphe nuclei terminate in deep cerebellar nuclei, cerebellar cortex, and spinal cord. Axons of neurons in the *rostral* raphe nuclei terminate in the thalamus, striatum, hypothalamus, NAc, neocortex, cingulate gyrus, cingulum, hippocampus, and amygdalae (Hornung, 2003). Thus, activation of this serotonin system has effects on large areas of the brain and seems to be involved in sexual behavior (Hull, Muschamp, & Sato, 2004).

Serotonin receptors are also located in the periphery of the body where serotonin acts on the smooth muscles of the vascular system of the genitals and other sexual organs to produce vasoconstriction and vasodilatation.

In CNS, 5‐HT has an inhibitory effect on sexual function (Croft, 2017). Antidepressants of the selective serotonin reuptake inhibitor class (SSRI) impair ejaculatory/orgasmic function and frequently inhibit erectile function, lubrification, and sexual interest. Interestingly, experimental lesions of a major source of 5‐HT to spinal cord, that is, nPG1, disinhibit the urethrogenital reflex (a model of sexual climax) and reflexive erections and penile anteroflexions, confirming the potential inhibitory role of serotonin on sexuality.

5‐HT receptors are highly heterogeneous, and they have been regrouped into seven different families. Whereas all the 5‐HT receptor subtypes are found postsynaptically, and appear to mediate an inhibitory effect on ejaculation, orgasm, and erection, only 5‐HT1A and 1B/D receptors are located presynaptically where they mediate the negative feedback of serotonin on its synaptic release (Giuliano & Clément, 2006). Stimulation of 5‐HT1A receptors, either systematically or in the MPOA, facilitates ejaculation, and systematic administration of a 5‐HT1A agonist reversed sexual satiety. Thus, it was suggested that 5‐HT1A agonists' beneficial effects may result from the stimulation of the inhibitory autoreceptors in the raphe nuclei, which would decrease 5‐HT levels. Otherwise, the facilitative effects of the 5‐HT1A agonist may be mediated in part through its increase in extracellular DA in the MPOA (Hull, 2011). Moreover, as 5HT1A receptors are found in the dorsal horn and dorsal gray matter commissure, it is likely that these receptors are involved in the spinal processing of sensory information to the brain also modulating the triggering ejaculation (Hull et al., 2004; Meston & Frohlich, 2000).

### 6.2 Dopamine

The role of DA in human sexuality is not completely understood yet, and most of our knowledge comes from animal models (Hull et al., 2004). DA in the striatum disinhibits pathways through which the cortex elicits movements: This neurotransmitter is released during copulation, but not during precopulatory exposure to a receptive female, suggesting that the ventral striatal DA (Dominguez & Hull, 2005) is important for motoric aspect of copulation, but not for sexual motivation. On the other hand, DA acts in the MPOA in order to promote male sexual behavior (Dominguez & Hull, 2005).

The fact that DA plays a pivotal role in human sexuality is partially confirmed considering that dopaminergic drugs have long been known to facilitate male sexual function clinically. In fact, it has been demonstrated that the classic DA agonist apomorphine is effective in treating erectile dysfunction with few side effects. From a physiological perspective, small increases in DA in MPOA disinhibit genital reflexes via a member of D2 receptors; moderate increases facilitate parasympathetically mediated erections and copulatory behavior via D1‐like receptor; and large increases promote sympathetically mediated ejaculation but inhibit erections (Dominguez, Gil, & Hull, 2006; van Furth, Wolterink, & Ree, 1995; Hull et al., 2004).

Interestingly, cocaine, during acute assumption, enhances dopamine activity by blocking the presynaptic autoreceptors and enhancing dopamine release itself, so that it is commonly seen as an “aphrodisiac” enhancing sexual desire, sexual performance, and pleasure perception (Jones, Garris, & Wightman, 1995; Venton, 2006). However, chronic cocaine users experience sexual disorders such as inhibited sexual arousal, diminished sexual desire, and delayed ejaculation mainly due to disruption of the dopaminergic system, which occurs after its continuous stimulation (Brown, Domier, & Rawson, 2005; Peugh & Belenko, 2001; Rawson, Washton, Domier, & Reiber, 2002). However, the precise effects of acute and chronic cocaine use are still unclear and it may be influenced by the context of the drug use (Leigh, 1990). Since cocaine is known to exert disinhibitory effects on behavior, many individuals could be driven to use this drug when they expect or seek to be engaged in sexual intercourse (Kopetz, Reynolds, Hart, Kruglanski, & Lejuez, 2010).

### 6.3 Norepinephrine

As a stress hormone, norepinephrine (NE) is released from the adrenal medulla into the blood and affects the body and parts of the brain where attention and responding actions are controlled. In fact, along with epinephrine, NE underlies the fight‐or‐flight response, directly increasing heart rate, triggering the release of glucose from energy stores, and increasing blood flow to the skeletal muscles. The noradrenergic neurons in the brain form a neurotransmitting system that, when activated, exerts effects on alertness, on arousal, and on the reward system. Anatomically, the noradrenergic neurons originate both in the locus coeruleus and in the lateral tegmental field as observed in animal studies. The axons of the neurons in the locus coeruleus act on adrenergic receptors in: amygdala, cingulate gyrus, cingulum, hippocampus, hypothalamus, neocortex, spinal cord, striatum, and thalamus. On the other hand, the axons of neurons of the lateral tegmental field act on adrenergic receptors in the hypothalamus (Calabrò & Bramanti, 2011). Therefore, NE release modulates different aspects of motivation with an “inverted U‐shaped curve” in which an optimal NE transmission supports an optimal level of behavior but in which a high amount of transmission disrupts behavior by producing a generalized fear response. Adrenergic activity plays a role in maintaining the penis in a flaccid state and producing detumescence. A1‐adrenergic receptors have been found in the human penile tissue, and inhibition of α1‐receptors produces an erection.

Studies reporting the effects of drugs that act on NE receptors indicate that the monoamine is important in male sexual function. As noted earlier, SSRIs produce a whole host of side effects, while the newer classes of antidepressant that act on NE neurotransmission (i.e., venlafaxine, duloxetine, mirtazapine) have been found to produce fewer side effects (Calabrò, Manuli, Portaro, Naro, & Quattrini, 2018). Taking into account that norepinephrine plays a facilitatory role in regulating human sexual behavior, the balancing of the negative effect of 5‐HT on sexuality (by increasing the brain norepinephrine levels) may justify the fact that the newer classes of antidepressant that act on NE neurotransmission have lower sexual dysfunction incidence than SSRI (Behrens, Berg, Jbabdi, Rushworth, & Woolrich, 2007; Clayton, Haddad, Iluonakhamhe, Ponce Martinez, & Schuck, 2014; Hull et al., 2004; Johannessen Landmark, Henning, & Johannessen, 2016; Montejo, Montejo, & Navarro‐Cremades, 2015).

Interestingly, administration of the α2 antagonist yohimbine stimulates penile erection via autonomic activation and can reverse the sexual inhibition that follows sexual exhaustion in male rats. Moreover, it is known that this drug could be useful in the treatment of erectile dysfunction and anorgasmia (Bancroft, 2002a; Meston & Frohlich, 2000).

### 6.4 Acetylcholine

Together with the vasoactive intestinal peptide, acetylcholine has been implicated in penile erection, which occurs when the smooth muscles of the corpus cavernosum relax permitting increased blood flow into the penile tissue. The human corpus cavernosum is innervated by cholinergic nerves and contains cholinergic receptors, suggesting endogenous activity of the Ach in the penile tissue. In addition, the cholinergic agent bethanechol has been reported to reverse antidepressant‐induced erectile and ejaculation difficulties (Bancroft, 2002a; Meston & Frohlich, 2000).

On the other hand, it is worth to note that yohimbine and bethanechol do not seem to be useful drugs clinically and we just mentioned them for sake of completeness.

### 6.5 Histamine

Histamine action of the ventromedial nucleus of the hypothalamus (VHM) in modulating sexual behavior is well known from studies conducted on rats. The H2 antagonists, cimetidine and ranitidine, have been shown to cause loss of libido and erectile failure, and it may partially result from reduction in uptake of testosterone (Calabrò & Bramanti, 2011). Peripherally, histamine is implicated in penile vasodilatation as its injection into the corpus cavernosum produces full or partial erection through the activation of H2 and H3 receptors (Zhou et al., 2007).

### 6.6 Opioids

Much of what is known about the role of opioids in the sexual response cycle comes from research on the effect of narcotics and agonists and antagonists of naturally occurring opioids such as endorphins, enkephalins, and dynorphins both on humans and animals. Indeed, it is well established that abuse of opioids leads to loss of libido, erectile dysfunction, and inability to reach orgasm (Gulliford, 1998; Holloway, Cornil, & Balthazart, 2004; Pfaus & Gorzalka, 1987). Withdrawal from opiate addiction in humans is characterized by increased frequency of morning erections, spontaneous ejaculation, and a slow return to sexual drive (Ouyang et al., 2012). Although the mechanism by which opiates affect sexual functioning is unclear, evidence suggests that the increase in opioid activity produces a decrease in the levels of circulating hormones, such as LH and testosterone with consequent sexual impairment (Gudin, Laitman, & Nalamachu, 2015; Seyfried & Hester, 2012).

### 6.7 Sex hormones

Sex hormones are essential for neural circuit development and sex‐specific behaviors. Male behaviors require both testosterone and estrogen, but it is still unclear how the two hormonal pathways intersect. Circulating testosterone activates the androgen receptors (ARs) and is also converted into estrogen in the brain via aromatase; it seems that this conversion, especially in the critical periods of brain development, is important for sexual behavior, differentiation, and orientation (Calabrò & Bramanti, 2011).

Within brain, testosterone binds to ARs, but can be converted in dihydrotestosterone (DHT) through the 5‐alpha‐reductase pathway and bind to AR, or to estradiol through the aromatase pathway and bind to estrogen receptors (ERs) which act to masculinize (increase male‐typical behaviors) and defeminize (reduce female‐typical responses) the behavioral development of male mammals. ARs are widely found in cerebral and subcortical regions of the human brain (MPOA, SNC, SDN, and INAH‐3, also known as the nucleus of homosexual orientation). Genetically influenced variations, or decreases in brain aromatase could produce feminization of male sexual preferences, in the absence of estradiol in these key neural regions (Bancroft, 2002a, 2002b).

Sexual hormones seem to play an important role in sexual arousal by ensuring cerebral integration between somatic and autonomic sexual systems. Indeed, they would contribute to the ascent of spinal sexual reflexes to the cerebral level with a consequent erogenization of genital stimulation via activation of autonomic centers. The level of sexual arousability is associated with a naturally occurring strong–weak (high–low) ratio of the two antagonist classes of sexual hormones, androgens and estrogens that, acting separately on each of the two antagonist axes (parasympathetic and sympathetic), could induce central agonist effects with sex differences in the sexual arousal and response sequence (Bancroft, 2002a; Lewis & Mills, 2004; Meston & Frohlich, 2000; Wu et al., 2009).

Androgens have a key role in both stimulating and maintaining sexual function in man; in particular, they are deemed critical for penile tissue development, growth, and maintenance of erectile function. There is growing insight that testosterone has profound effects on the tissues of the penis involved in the mechanism of erection as androgen deprivation causes penile tissue atrophy, changes in dorsal nerve structure, changes in endothelial morphology, reduced trabecular smooth muscle content, and alterations in extracellular matrix. In addition, testosterone is involved in clitoral engorgement and genital lubrication (Nappi et al., 2003).

It is believed that the level of testosterone required for sexual interest and activity in adult males is lower than normal males' circulating levels of testosterone. Therefore, variability in testosterone levels above this threshold level, or exogenously induced testosterone changes above this level, would not be expected to influence sexual interest or behavior. On the other hand, it is clear that loss of testosterone is associated with loss of libido both in men and in women (Andersen & Tufik, 2006). Lower testosterone levels in women due to hypopituitarism and premature ovarian failure are related to a decreased sexual desire typical in hypoactive sexual disorder and female sexual arousal disorder (Kingsberg & Rezaee, 2013). Testosterone therapy is currently used to manage hypoactive sexual desire disorders (Abdallah & Simon, 2007).

The physiological underpinning of libido seems to depend on androgenic actions on the paraventricular nucleus of the hypothalamus, an integration center between the central and peripheral autonomic nervous systems that, despite its projections to many important sexual brain areas, controls penile erection (Morales et al., 2004, 2009).

On the other hand, it is likely that estrogens have little direct influence on sexual desire as, in men, relatively high levels of exogenous estrogen have been somewhat effective in inhibiting sexual desire in sexual offenders.

In contrast, prolactin (PRL) provides the body with sexual gratification after sexual acts. Sexual arousal and stimulation per se do not alter prolactin levels significantly, but changed postorgasmic levels might be of crucial interest for the interpretation of refractoriness and loss of sexual drive. It has been recently hypothesized that PRL may represent a negative feedback mechanism whereby this hormone may modify the activity of dopaminergic neurons in the CNS, especially in the nigrostriatal and mesolimbocortical system and the MPOA, controlling different aspects of sexual behavior. Interestingly, a single case study showed that a multiorgasmic male had a striking absence of orgasm‐induced PRL secretion that paralleled an extremely short refractory period. Finally, high PRL blood levels are suspected to be responsible for impotence and loss of libido (Krüger et al., 2003; Krüger, Haake, Hartmann, Schedlowski, & Exton, 2002).

## 7 AUTHORS' REMARK, CLINICAL IMPACT, AND CONCLUSIONS

In the present scoping review, we aimed at characterizing the neural anatomo‐physiological bases of human sexuality, describing how the brain helps to initiate and maintain sexual arousal and desire, ultimately leading to the orgasmic phase. Although the knowledge on human sexual behavior is of growing importance, also considering its social and personal impact, it is still not clear whether this topic is fully addressed or quite neglected in clinical practice, often due to a lack of education and training in human sexuality during medical school and the entire course of life. Nonetheless, the knowledge of the neural correlates and function of human sexual behavior is fundamental to better manage patients, especially those with neurological disorders (Calabrò et al., 2014; Calabró, Gervasi, & Bramanti, 2011; Calabro, Marino, & Bramanti, 2011). Indeed, brain and spinal cord injury, as well as peripheral disorders, may frequently affect sexual function and therefore lead to a poor quality of life. Too often, physicians believe that sexuality is not as important as the injury or illness that brought the patient to the medical team. The quality of personal relationships, sexual ones in particular, exerts great impact on a patient's self‐esteem and support network. The multiple physical, psychological, and emotional changes that may occur after a catastrophic injury, or as a result of a congenital disability or chronic illness, must be addressed not only in the context of the patient, but also of the patient's support system. Providing healthcare professionals with information concerning sexual behavior may overcome useless and sometimes dangerous barriers and improve patient therapeutic alliance, since sexual well‐being is nowadays considered one of the most important aspects of one's quality of life.

## CONFLICT OF INTEREST

The authors declare they have no conflict of interest nor financial support.

## Data Availability

Research data are not shared.
